# A non-invasive prediction model for coronary artery stenosis severity based on multimodal data

**DOI:** 10.3389/fphys.2025.1592593

**Published:** 2025-06-02

**Authors:** Jiyu Zhang, Jiatuo Xu, Liping Tu, Tao Jiang, Yu Wang, Jijie Xu

**Affiliations:** ^1^ College of Traditional Chinese Medicine, Shanghai University of Traditional Chinese medicine, Shanghai, China; ^2^ Shanghai Baoshan Hospital of Integrated Traditional Chinese and Western Medicine, Shanghai, China

**Keywords:** coronary artery disease, multimodal prediction, deep learning approaches, cardiovascular risk assessment, machine learning for disease risk stratification

## Abstract

**Introduction:**

Coronary artery disease (CAD) diagnosis currently relies on invasive coronary angiography for stenosis severity assessment, carrying inherent procedural risks. This study develops a transformer-based multimodal prediction model to provide a clinically reliable non-invasive alternative. By integrating heterogeneous biomarkers including facial morphometrics, cardiovascular waveforms and biochemical indicators, we aim to establish an interpretable framework for precision risk stratification.

**Methods:**

The study utilized a transformer-based architecture integrated with residual modules and adaptive weighting mechanisms. Multimodal data, including facial features, lip and tongue images, pulse and pressure wave amplitudes, and laboratory indicators, were collected from 488 CAD patients. These data were processed and analyzed to predict the severity of coronary artery stenosis. The model’s performance was evaluated using both internal and external validation datasets.

**Results:**

The proposed model demonstrated high predictive accuracy, achieving over 90% accuracy in assessing coronary artery stenosis risk on the training dataset. External validation on real-world data further confirmed the model’s robustness, with an accuracy of 85% on the validation set. The integration of multimodal data and advanced architectural components significantly enhanced the model’s performance.

**Conclusion:**

This study developed a non-invasive, transformer-based multimodal prediction model for assessing coronary artery stenosis severity. By combining diverse data sources and advanced machine learning techniques, the model offers a clinically viable alternative to invasive diagnostic methods. The results highlight the potential of multimodal data integration in improving CAD diagnosis and patient care.

## 1 Introduction

Coronary artery disease (CAD) is a prevalent chronic cardiovascular condition worldwide, characterized by consistently high incidence and mortality rates. Epidemiological studies across different countries have identified CAD as a high-risk disease (Roth et al., 2020; Duan et al., 2024a; SU Wei, 2024; Tian et al., 2024). In recent years, advancements in coronary computed tomography angiography (CTA) have established it as a critical diagnostic tool for assessing coronary artery stenosis, becoming one of the key diagnostic standards for CAD. However, due to the invasive nature of CTA, it is not suitable for all patients. In clinical practice, patients diagnosed with significant stenosis via CTA often undergo percutaneous coronary intervention (PCI) to restore blood flow. However, complications such as calcification, bifurcation issues, and multivessel disease can arise postoperatively, causing additional strain on the patient’s health (Iftikhar et al., 2024). Therefore, there is a pressing need for a non-invasive diagnostic method for coronary artery stenosis. Such a method could serve as an alternative to CTA for patients who are unsuitable candidates and provide auxiliary recommendations for stenosis severity. Furthermore, it could reduce the need for exploratory PCI procedures, thereby minimizing unnecessary vascular damage.

In recent years, advancements in deep learning and artificial intelligence have accelerated the adoption of non-invasive diagnostic techniques (Le et al., 2024; Liu et al., 2024a). In the domain of CAD, AI has been successfully applied to areas such as depression in CAD patients (Hou et al., 2024), atrial fibrillation prediction (Jian et al., 2024), genetic risk estimation, and hemodynamic modeling (Mishra et al., 2024; Rasmussen et al., 2024). Studies incorporating imaging data for CAD prediction have also shown promise. For instance, research has indicated that facial features of patients are associated with an increased risk of coronary artery disease. A deep learning algorithm developed for CAD prediction based on facial photographs achieved a sensitivity of 0.80, specificity of 0.54, and an area under the curve (AUC) of 0.730 (Lin et al., 2020). This suggests that facial photographs can, to some extent, predict CAD.

Additionally, other studies have demonstrated the predictive value of pulse pressure wave velocity (PWV) for CAD, particularly showing stronger associations in males compared to females (Chiha et al., 2016; Park et al., 2019; Hametner et al., 2021). Tongue features have also been employed in CAD diagnostics, improving model performance when included (accuracy = 0.760, precision = 0.773, AUC = 0.786), indicating the feasibility of using tongue characteristics for CAD detection (Duan et al., 2024b). In the context of non-invasive diagnostic techniques incorporating facial, tongue, and pulse features, prediction models for pulmonary diseases have also achieved promising results (AUC = 0.825 for the best-performing comprehensive syndrome diagnosis model) (Zhou et al., 2023).

Meanwhile, in recent years, there have been studies focusing on the prognostic prediction of coronary heart disease (CHD) using multimodal data, including the use of different types of clinical data to predict the prognosis of coronary artery disease (Xu et al., 2024), the combination of cardiovascular imaging techniques and biomarkers for the diagnosis of elderly patients with CHD (Liu et al., 2024b), and the use of multimodal laboratory indicators for the early prediction of cardiovascular and cerebrovascular diseases (Wang et al., 2024). Although current research demonstrates that multimodal data fusion is superior to single data in the diagnosis of CHD, there is still room for improvement in the research methods of multimodal data fusion.

Motivated by these findings, we aim to integrate clinical indicators, CAD risk factors, and multimodal data—including facial images, tongue images, and pulse data—into a unified multimodal representation learning framework (Zhang et al., 2019). This approach allows us to construct a multimodal disease risk prediction model (Zhou et al., 2023; Mishra et al., 2024), which combines image features, numerical data, and pressure wave values to enable non-invasive prediction of CAD risk. The model incorporates well-established CAD risk factors, such as age, smoking status, systolic blood pressure, diabetes history, and total cholesterol levels (Khera et al., 2016; Di Angelantonio et al., 2019). By training a machine learning model with these risk factors and clinical indicators, we aim to develop a binary classification model that predicts whether coronary artery stenosis exceeds 75%, Adaptive Weighted Cardiovascular Occlusion Prediction Model (AWCOP_Model). Patients with stenosis greater than 75% are generally considered candidates for PCI(Boden et al., 2007; Amsterdam et al., 2014). This model would thus assist in assessing high-risk cases requiring surgical intervention. Through the construction of multimodal data models, new avenues can be provided for the non-invasive prediction of coronary artery occlusion in coronary heart disease, offering additional value to clinicians when CTA diagnosis is unavailable.

## 2 Materials and methods

### 2.1 Case collection

#### 2.1.1 Study subjects

**FIGURE 1:**
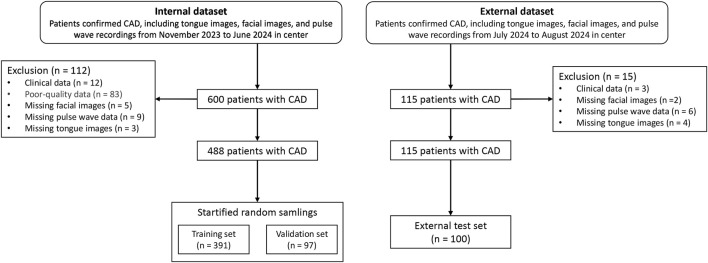
Data collection flow chart.

The study cohort consisted of patients hospitalized in the Cardiology Department of Shanghai Baoshan District Integrated Traditional Chinese and Western Medicine Hospital between October 2023 and July 2024. Among the participants, 243 patients were diagnosed with coronary artery stenosis ≥75% via coronary CTA, while 214 patients had stenosis <75%. External validation data were collected in August 2024 from additional patients hospitalized in the same department. Clinical data for the patients are summarized in Table 1. The case screening process is shown in Figure 1.

**TABLE 1:** Clinical significance of pulse acquisition equipment.

Parameter	Clinical significance
h1	Reflects the compliance of large arteries and the ejection function of the left ventricle
h3	Reflects arterial elasticity and the state of peripheral resistance
h4	Reflects peripheral arterial resistance and aortic valve function
h5	Related to the compliance of large arteries and aortic valve function
t1	Corresponds to the rapid ejection phase of the left ventricle
t4	Corresponds to the systolic period of the left ventricle
t5	Corresponds to the diastolic period of the left ventricle
t	Corresponds to the cardiac cycle of the left ventricle
h3/h1	Reflects the state of peripheral resistance and vascular wall compliance
h1/t1	Reflects the strength of cardiovascular function
h4/h1	Reflects the level of peripheral vascular resistance
w1/t	w1 represents the top one-third of the primary wave, reflecting the duration of elevated aortic pressure
w2/t	w2 represents the top one-fifth of the primary wave, reflecting the duration of elevated aortic pressure
t1/t	t1/t reflects the strength of cardiac ejection function
t4/t5	Reflects the speed of heart rate

The study used the validation dataset (n = 97) to train the parameters, which served as the model’s Internal Data results, while the external test set (n = 100) was used as the results of the external dataset.

This study has been reviewed and approved by the Ethics Committee of Shuguang Hospital Affiliated to Shanghai University of Traditional Chinese Medicine, with Registration Number 2020-916-125. It complies with the Helsinki Declaration of the World Medical Association guidelines. All participants in this study provided written informed consent prior to their inclusion. The objectives, procedures, potential risks, and anticipated benefits of the study were comprehensively communicated to each participant. The research was conducted in full compliance with the principles of the Declaration of Helsinki, ensuring that the rights, safety, and welfare of all participants were safeguarded throughout the study. At the same time, the obtained patient pulse pressure waveform data, facial images, and tongue images were used with the patient’s consent, and efforts were made to protect any privacy that may be involved.

#### 2.1.2 Diagnostic criteria

The diagnostic criteria for CAD were based on the ninth edition of Internal Medicine, published by People’s Medical Publishing House, which defines acute and chronic CAD. The classification includes chronic CAD—encompassing stable angina, ischemic cardiomyopathy, and latent CAD—and acute coronary syndrome (ACS), which includes unstable angina, non-ST-segment elevation myocardial infarction, and ST-segment elevation myocardial infarction (Ge et al., 2018).

#### 2.1.3 Inclusion criteria

Patients were included in the study if they met the following criteria:

1. Diagnosed with CAD according to the established diagnostic criteria.
2. Exhibited typical chest pain symptoms, such as paroxysmal angina or compressive pain.
3. Showed diminished heart sounds on auscultation.
4. Had electrocardiogram (ECG) findings of ST-segment abnormalities.
5. Met the diagnostic criteria outlined in the 2019 ESC Guidelines for the Diagnosis and Management of Chronic Coronary Syndromes published by the European Society of Cardiology (Knuuti et al., 2020).

#### 2.1.4 Exclusion criteria

Patients were excluded if they met any of the following criteria:

1. Did not meet the CAD diagnostic criteria.
2. Were younger than 20 years or older than 85 years.
3. Suffered from malignant tumors or critical illnesses.
4. Were pregnant or breastfeeding women.
5. Had incomplete clinical or imaging data.

### 2.2 Data collection equipment

Tongue and facial images were collected using the TFDA-1 digital tongue and facial diagnostic instrument, developed by the Shanghai University of Traditional Chinese Medicine. Data analysis for tongue images was performed using the university’s proprietary Traditional Chinese Medicine Tongue Diagnosis Analysis System (TDAS) V2.0. Recent studies have summarized the classification and typology of tongue features, demonstrating the reliability of such diagnostic tools (Jiatuo et al., 2024). This device was specifically used for stable and standardized collection of tongue and facial images.

**FIGURE 2:**
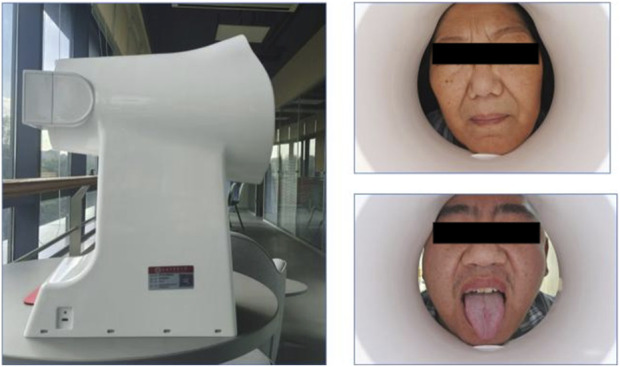
Tongue diagnosis and face-to-face diagnosis collection equipment.

The imaging parameters were set as follows: Shutter Speed: 1/125, seconds Aperture: F6.3, ISO Sensitivity: ISO 200, Standardized tongue image analysis was conducted using the TDAS-3.0 software, which quantifies tongue features based on predefined parameters. This system ensures consistency and objectivity in the analysis of tongue and facial images (Figure 2).

**FIGURE 3:**
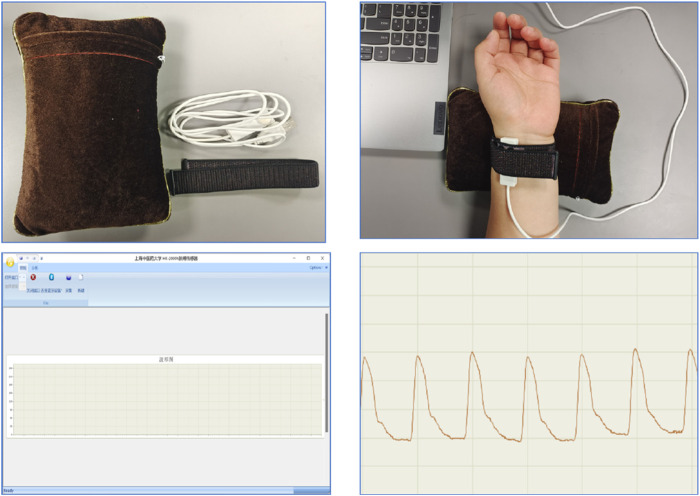
PDA-1 pulse equipment.

Pulse diagnostic indicators: including pulse intensity indicators, time indicators, ratio indicators (h1, h3, h4, h5, t, t1, t4, t5, h3/h1, h1/t1, h4/h1, t1/t, t4/t5, w1/t, w2/t). See Table 2 for the significance of the actual parameters of the instrument. The equipment parameters and acquisition process are shown in Figure 3 and Table 1.

**TABLE 2:** Model module parameters.

Category	Submodule	Input dimensions	Output dimensions	Description
Tongue image	Conv2D	(N, 3, H, W)	(N, 64, H, W)	Convolution operation (3 × 3 kernel)
	BatchNorm2D	(N, 64, H, W)	(N, 64, H, W)	Batch normalization
	ReLU	(N, 64, H, W)	(N, 64, H, W)	Activation function
	MaxPool2D	(N, 64, H, W)	(N, 64, H/2, W/2)	Max pooling (2 × 2 kernel)
	Residual Block × 4	(N, 64, H/2, W/2)	(N, 64, H/2, W/2)	Four-layer residual block
	AdaptiveAvgPool2D	(N, 64, H/2, W/2)	(N, 64, 1, 1)	Adaptive average pooling
	FC (Fully Connected)	(N, 64)	(N, 128)	Fully connected layer
	Self-Attention Mechanism	(N, 128)	(N, 128)	Extracts key features and enhances correlations
Face image	Conv2D	(N, 3, H, W)	(N, 64, H, W)	Convolution operation (3 × 3 kernel)
	BatchNorm2D	(N, 64, H, W)	(N, 64, H, W)	Batch normalization
	ReLU	(N, 64, H, W)	(N, 64, H, W)	Activation function
	MaxPool2D	(N, 64, H, W)	(N, 64, H/2, W/2)	Max pooling (2 × 2 kernel)
	Residual Block × 4	(N, 64, H/2, W/2)	(N, 64, H/2, W/2)	Four-layer residual block
	AdaptiveAvgPool2D	(N, 64, H/2, W/2)	(N, 64, 1, 1)	Adaptive average pooling
	FC (Fully Connected)	(N, 64)	(N, 128)	Fully connected layer
	Self-Attention Mechanism	(N, 128)	(N, 128)	Extracts key features and enhances correlations
Pulse wave	LSTM	(seq_len, N, 1)	(seq_len, N, 64)	Long Short-Term Memory network (LSTM unit)
	FC (Fully Connected)	(N, 64)	(N, 128)	Fully connected layer from LSTM output
Clinical data	FC (Fully Connected)	(N, 15)	(N, 128)	Fully connected layer for clinical data input
Adaptive weight	Weighted Summation (Learnable Weights)	(N, 128) (per branch)	(N, 512)	Fusion of tongue, face, pulse, and clinical data
	FC (Fully Connected)	(N, 512)	(N, 2)	Final classification output (whether vascular blockage >75%)

## 3 Model construction

### 3.1 Model framework

**FIGURE 4:**
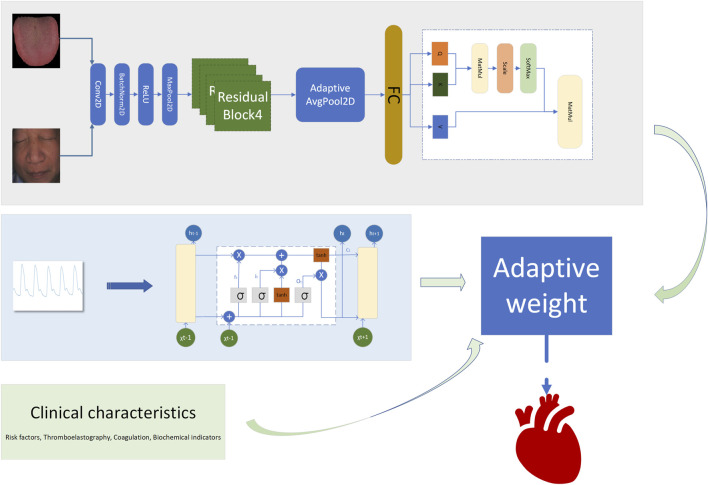
Adaptive weighted cardiovascular occlusion prediction model (AWCOP_Model).

A fusion decision network model was constructed based on clinical data, facial images, tongue images, and radial pulse wave data (as illustrated in Figure 4, Table 2). Facial and tongue images were processed using 3 × 3 convolutional kernels followed by max-pooling operations before being passed through four residual modules. Residual modules, a well-established component in deep learning, have been proven to significantly enhance image classification performance in neural networks, achieving optimal results on public datasets such as ImageNet (He et al., 2016). Recent studies have also highlighted the broad application prospects of residual modules in various domains (Shafiq and Gu, 2022).

In the proposed model, we incorporated a transformer-based self-attention mechanism after the residual modules to further improve performance. The self-attention mechanism has been demonstrated to effectively capture multi-angle information and combine semantic features, thereby enhancing the model’s representational power and accuracy. Additionally, the residual modules improve gradient propagation when combined with the self-attention mechanism, mitigating issues such as vanishing or exploding gradients during training (Vaswani et al., 2017; Dosovitskiy et al., 2020).

Radial artery pulse wave data were collected using a PDA-1 pressure sensor-based pulse diagnosis instrument. During data acquisition, participants maintained a sitting or supine position, with their wrist resting on a pulse pillow. The sensor probe was placed on the radial artery of either the left or right hand. Once a stable pulse waveform was observed, data were collected for 30 s and saved. The raw data underwent preprocessing steps, including smoothing and noise removal, to eliminate artifacts and data drift. The pressure waveform data points were sampled at a frequency of 1/50 s, and a stable waveform segment of 6 s in duration was extracted.

For processing the pulse wave signal, we employed the long short-term memory (LSTM) mechanism (Hochreiter and Schmidhuber, 1997), which is particularly effective for sequential data processing. LSTM has been validated for use in disease diagnosis applications, demonstrating its robustness and reliability in similar tasks (Saif et al., 2024).

By integrating these components—convolutional layers, residual modules, self-attention mechanisms, and LSTM processing—we constructed a multimodal fusion model capable of leveraging diverse data sources to predict the severity of coronary artery stenosis.

### 3.2 Adaptive weight algorithm

To enhance the model’s performance, we introduced an adaptive weight (adaptive_weight) mechanism into the final output layer. This algorithm dynamically adjusts the contribution of each modality (tongue, face, pulse, and laboratory data) to the final prediction by automatically allocating weights based on their individual output significance. The concept of fusing multimodal data at the backend through adaptive weighting has been explored in recent studies (Huq and Pervin, 2022; Wang et al., 2023). Additionally, we compared the performance of our adaptive weight module with other weight allocation methods to evaluate its effectiveness.

The adaptive weight algorithm addresses discrepancies in evaluation caused by differences in data dimensions during multimodal fusion at the backend. Through weight initialization and spatial alignment mapping, the algorithm ensures consistent weighting across modalities, mitigating biases resulting from dimensional variations. Specifically, the process aligns data from multiple modalities onto a shared temporal or feature space, which is critical when combining or comparing data from heterogeneous sources. This alignment step enhances classification consistency and improves the accuracy of the model’s predictions.

**FIGURE 5:**
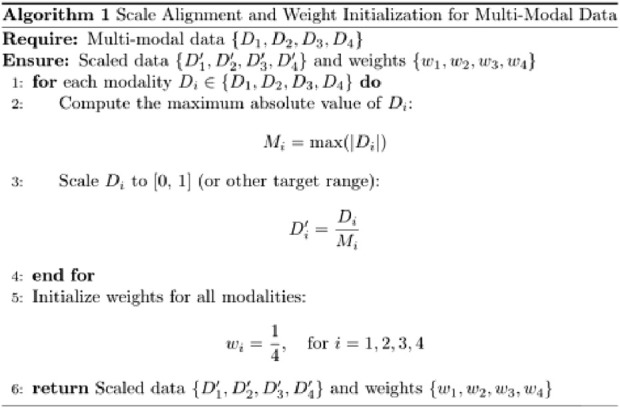
Scale alignment and weight initialization for multi-modal data.

**FIGURE 6:**
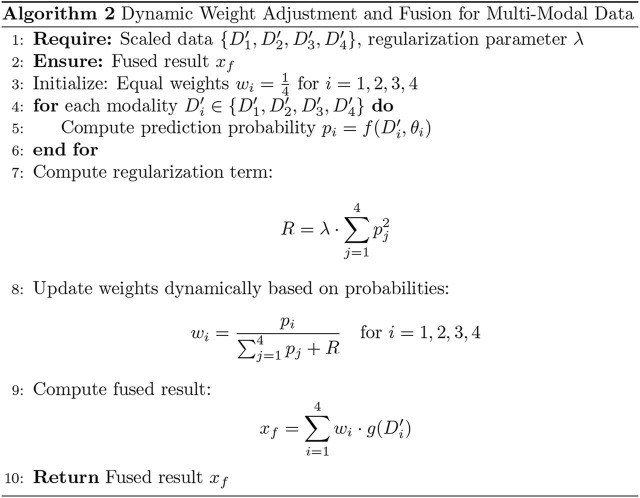
Dynamic weight adjustment and fusion for multi-modal data.

By leveraging this adaptive weight mechanism, the model can autonomously learn the relative importance of each modality and assign appropriate weights during the final decision-making process. This approach not only improves multimodal integration but also optimizes the model’s ability to utilize complementary information across modalities (as shown in Figures 5, 6). The R in Figure 6 represents the regularization term.

### 3.3 Heatmap algorithm

**FIGURE 7:**
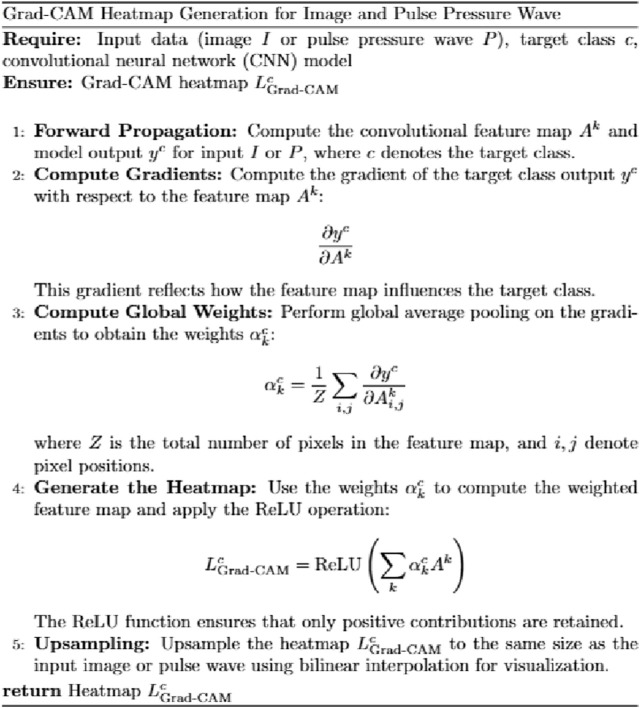
Grad-CAM heatmap generation for image and pulse pressure wave.

The heatmap visualization in our model utilizes the Grad-CAM (Gradient-weighted Class Activation Mapping) approach (Selvaraju et al., 2020; Li et al., 2024). Grad-CAM is well-suited for multimodal tasks and does not require retraining of the model, making it an efficient tool for understanding the learning process of convolutional neural networks (CNNs) in image classification tasks. By combining gradient information with feature maps, Grad-CAM highlights the key regions of input data that contribute most significantly to the model’s predictions, thereby providing interpretability for the internal mechanisms of the deep learning model (Figure 7).

For the visualization of pulse wave data heatmaps, we marked the top 20% of points contributing to the prediction using black dots. This approach helps identify the primary regions of focus within the pulse waveform that the model considers critical for its decision-making process.

By employing Grad-CAM across the different modalities (e.g., tongue and facial images, pulse wave data), the algorithm enables a clearer understanding of how the model integrates and prioritizes information from each input source. This interpretability is crucial for validating the model’s behavior and gaining insights into its decision-making logic, particularly in clinical applications where trust in AI predictions is paramount.

### 3.4 Clinical feature importance screening

The clinical dataset in this study consisted of 50 dimensions, including patients’ basic physiological indicators, thromboelastography results, and coagulation parameters. To prevent overfitting and reduce the complexity of clinical data dimensions, random forest importance analysis was employed to evaluate the significance of each feature. The top 15 most important clinical features were selected and incorporated into the model training process to optimize its performance.

### 3.5 Machine learning

To compare the performance of the proposed model with traditional machine learning algorithms, we tested five classical methods: Logistic Regression (LR), Random Forest (RF), Support Vector Machine (SVM), K-Nearest Neighbors (KNN), and Extreme Gradient Boosting (XGBoost). Radial pulse wave parameters were extracted using the PDA-1 pulse pressure wave analysis system, while tongue and facial image parameters were collected using the TFDA-1 tongue and facial diagnostic instrument. These extracted parameters, combined with clinical data, formed four input dimensions—pulse, tongue, face, and clinical data—which were used for training and testing the machine learning models.

The performance of the models was evaluated using four standard metrics: AUC (Area Under the Curve): Reflects the overall performance of the model across different classification thresholds. ACC (Accuracy): Indicates the overall classification accuracy. F1-Score: Balances Precision and Recall to provide a comprehensive performance metric. Recall: Measures the ability of the model to correctly identify positive cases. This evaluation framework allowed for a systematic comparison of the proposed model with traditional machine learning approaches, ensuring the robustness and reliability of the multimodal prediction framework for clinical applications.

## 4 Results

### 4.1 Laboratory index screening

As shown in Table 3, the significantly different indicators between the two groups include Fibrinogen, D-Dimer, Fibrin (ogen) Degradation Products, and Myoglobin, suggesting that vascular obstruction is associated with increased fibrinogen levels, which may impact the vasculature.

**TABLE 3:** Analysis of the difference in the degree of coronary artery occlusion.

Classification	Vascular obstruction <75% (n = 214)	Vascular obstruction ≥75% (n = 243)	U/χ2	p
Hypertension	96/48 (male/female)	219/94 (male/female)	0.360	0.549
Diabetes mellitus	220/86 (male/female)	95/56 (male/female)	3.400	0.065
Alcohol consumption history	245/107 (male/female)	70/35 (male/female)	0.203	0.653
Family medical history	224/106 (male/female)	91/36 (male/female)	0.447	0.504
Age	66.00 (61.00, 76.00)	68.00 (61.25, 74.75)	914.500	0.787
Body mass index (BMI)	24.98 (23.44, 27.13)	24.67 (23.22, 26.65)	1,051.000	0.420
Clotting time (min)	5.00 (4.30, 6.00)	5.05 (4.25, 6.17)	932.500	0.899
Blood clot formation rate (min)	1.70 (1.40, 2.10)	1.50 (1.20, 2.08)	1,067.500	0.347
Blood clot aggregation rate (deg)	66.70 (63.60, 69.90)	68.95 (62.12, 71.83)	824.500	0.324
Blood clot dissolution rate (%)	0.00 (0.00, 0.10)	0.00 (0.00, 0.10)	902.000	0.667
Blood clot lysis percentage (%)	0.00 (0.00, 0.10)	0.00 (0.00, 0.10)	847.000	0.338
Coagulation comprehensive index	1.30 (−0.50, 2.40)	1.65 (−1.20, 3.40)	830.000	0.346
Platelet aggregation (AA) (mm)	13.00 (9.10, 22.10)	12.35 (9.57, 31.18)	898.500	0.691
Inhibition rate AA (%)	89.60 (75.80, 99.30)	93.10 (72.38, 98.65)	912.500	0.774
Platelet aggregation (ADP) (mm)	32.70 (22.20, 46.20)	33.10 (21.62, 45.25)	940.500	0.949
Inhibition rate (%)	53.00 (37.60, 75.40)	64.55 (48.58, 71.93)	877.000	0.570
Whole blood reduction rate 200 (1/s) mPa·s	3.99 (3.45, 4.72)	4.08 (3.65, 4.82)	851.000	0.438
Whole blood reduction rate 30 (1/s) mPa·s	4.94 (4.32, 5.78)	4.86 (4.38, 6.08)	888.000	0.631
Whole blood reduction rate 5 (1/s) mPa·s	7.42 (6.38, 8.61)	7.27 (6.51, 9.43)	840.000	0.388
Whole blood reduction rate 1 (1/s) mPa·s	14.02 (11.91, 17.78)	13.93 (12.06, 19.40)	876.500	0.567
Plasma viscosity (mPa·s)	1.55 (1.45, 1.65)	1.60 (1.45, 1.66)	856.500	0.462
Whole blood high shear relative index	2.67 (2.28, 2.98)	2.77 (2.40, 3.08)	801.000	0.241
Whole blood low shear relative index	9.21 (7.73, 11.08)	10.19 (7.66, 11.49)	833.000	0.358
Red blood cell Aggregation index	3.58 (3.03, 4.23)	3.56 (3.20, 4.10)	949.000	0.913
Casson viscosity (mPa·s)	3.33 (2.93, 3.76)	3.35 (3.12, 3.76)	850.000	0.434
Prothrombin time (s)	11.40 (11.00, 12.10)	11.35 (10.88, 12.00)	961.000	0.927
International normalized ratio (INR)	0.99 (0.95, 1.06)	0.98 (0.94, 1.05)	958.500	0.943
Partial thromboplastin time (PTT)	27.10 (23.50, 29.60)	26.35 (22.60, 27.40)	1,110.000	0.202
Fibrinogen (g/L)	2.69 (2.38, 3.08)	3.21 (2.74, 3.99)**	570.000	0.003
Thrombin time (s)	16.50 (16.00, 17.50)	16.95 (15.50, 18.18)	910.000	0.759
D-Dimer measurement (μg/mL)	0.29 (0.16, 0.48)	0.43 (0.27, 0.64)*	676.000	0.030
Fibrin (ogen) degradation products (μg/mL)	2.50 (1.70, 2.50)	2.50 (2.50, 3.18)*	694.500	0.040
Antithrombin III activity measurement (%)	89.10 (81.30, 97.10)	91.20 (84.83, 99.80)	796.500	0.227
Total cholesterol (mmol/L)	3.94 (3.12, 5.06)	4.06 (3.56, 4.88)	903.000	0.718
Triglycerides (mmol/L)	1.19 (0.84, 1.72)	1.38 (1.04, 1.70)	834.000	0.363
High-density lipoprotein (mmol/L)	1.11 (0.93, 1.28)	1.03 (0.88, 1.30)	963.500	0.911
Low-density lipoprotein (mmol/L)	2.29 (1.73, 3.00)	2.42 (2.04, 2.93)	828.500	0.340
Glycated hemoglobin (%)	6.10 (5.70, 6.80)	5.95 (5.62, 7.25)	951.000	0.991
Creatine kinase (U/L)	71.00 (61.00, 96.00)	86.00 (68.50, 98.50)	771.500	0.159
Creatine kinase Isoenzyme (U/L)	1.11 (0.84, 1.52)	1.30 (0.91, 1.88)	754.500	0.123
Myoglobin (ng/mL)	38.14 (31.46, 48.52)	48.34 (40.38, 62.29)**	570.000	0.003

*p < 0.05, **p < 0.01.

### 4.2 Laboratory index screening based on machine learning

**FIGURE 8:**
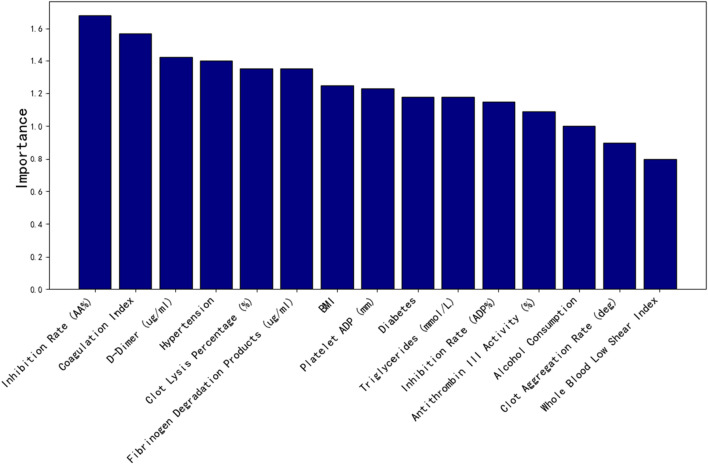
Top 15 clinical data importance.

As shown in Figure 8, the top 15 important features screened by random forest factors are: Inhibition rate (AA%), Coagulation index, D-Dimer (ug/mL), Hypertension, Clot lysis percentage (%), Fibrinogen degradation products (ug/mL), BMI, Platelet ADP (mm), Diabetes, Triglycerides (mmol/L), Inhibition rate (ADP%), Antithrombin III activity (%),Alcohol consumption, Clot acceleration time (deg), Whole blood low shear index.

### 4.3 AWCOP and machine learning assessment

**FIGURE 9:**
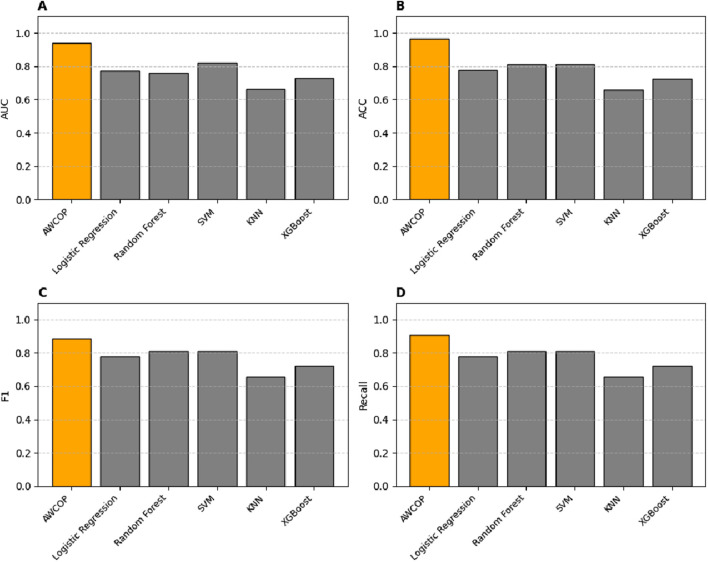
**(A)** Different models of AUC; **(B)** Different models of ACC; **(C)** Different models of F1; **(D)** Different models of Recall.

As shown in Table 4 and Figure 9, the evaluation performance of AWCOP is better than that of machine learning models. The AWCOP model performed best (AUC = 0.940, ACC = 0.964, F1 = 0.884, recall = 0.905). In the machine learning model, SVM performs best (AUC = 0.822, ACC = 0.811, F1 = 0.811, recall = 0.811).

**TABLE 4:** Comparison of evaluation parameters of different models (Internal date).

Model	AUC	ACC	F1	Recall
AWCOP	0.940	0.964	0.884	0.905
Logistic regression	0.772	0.778	0.778	0.778
Random forest	0.760	0.811	0.811	0.811
SVM	0.822	0.811	0.811	0.811
KNN	0.662	0.659	0.659	0.659
XGBoost	0.729	0.724	0.724	0.724

### 4.4 Ablation experiment of model module

Table 5 compares the performance of various models on both the internal dataset and the external dataset, evaluated using five metrics: AUC, ACC, F1, Recall, and Accuracy. Among the models, the LSTM + RESNET + ATTENTION model performed better on the internal dataset, achieving an AUC of 0.940, ACC of 0.964, F1 of 0.884, Recall of 0.905, and Accuracy of 0.863. On the external dataset, this model also demonstrated superior performance, with an AUC of 0.811, F1 of 0.799, and Recall of 0.803. In comparison, the RESNET + ATTENTION model ranked second in performance, achieving an AUC of 0.908, ACC of 0.934, and F1 and Recall values of 0.912 on the internal dataset. On the external dataset, it achieved an AUC of 0.864, F1 of 0.723, and Recall of 0.823. Single-component models, such as Attention, RESNET, and LSTM, showed relatively lower performance, with AUC values of 0.798, 0.798, and 0.803, respectively. This indicates that the multimodal integration of LSTM, RESNET, and Attention mechanisms effectively enhances the model’s performance, particularly in handling both internal and external datasets.

**TABLE 5:** Ablation results of AWCOP model module (Internal date).

Model	Internal date				External date			
	AUC	ACC	F1	Recall	AUC	ACC	F1	Recall
LSTM + RESNET + ATTENTION	0.940	0.964	0.884	0.905	0.811	0.863	0.799	0.803
RESNET + ATTENTION	0.908	0.934	0.912	0.912	0.864	0.834	0.723	0.823
LSTM + ATTENTION	0.876	0.887	0.783	0.873	0.809	0.783	0.698	0.871
LSTM + RESNET	0.923	0.943	0.817	0.956	0.829	0.834	0.814	0.815
ATTENTION	0.798	0.673	0.623	0.718	0.716	0.689	0.672	0.711
RESNET	0.798	0.709	0.734	0.723	0.801	0.798	0.754	0.661
LSTM	0.803	0.801	0.798	0.739	0.795	0.756	0.871	0.856

### 4.5 Parameter comparison of the different learning rates of the model

**FIGURE 10:**
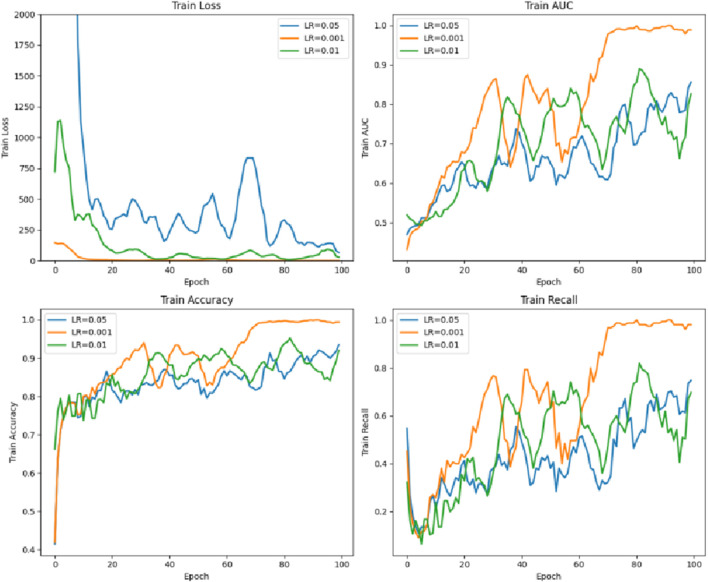
Comparison of the training parameters for the different learning rates.

As shown in Figure 10, the learning rate LR = 0.001 is the best, while LR = 0.05 is poor.

### 4.6 Data ablation experiment

As shown in Table 6, in the model data ablation analysis, the AUC, ACC, F1, and Recall values for the internal data outperform those of the external data. The training accuracy of laboratory and tongue images is the lowest, and the laboratory performance of tongue, surface and pulse data is the best. Internal training data (ACC = 0.964, AUC = 0.940, F1 = 0.884, Recall = 0.905) and external data training results were (ACC = 0.863, AUC = 0.811, F1 = 0.799, Recall = 0.803).

**TABLE 6:** Results of the ablation experiments for the model data.

Date type	Internal date				External date			
	AUC	ACC	F1	Recall	AUC	ACC	F1	Recall
Lab + tongue	0.799	0.925	0.712	0.619	0.698	0.657	0.709	0.594
Lab + face	0.737	0.885	0.579	0.524	0.611	0.709	0.689	0.734
Lab + pulse	0.863	0.950	0.816	0.738	0.861	0.825	0.635	0.881
Lab + tongue + face	0.930	0.964	0.881	0.881	0.855	0.856	0.829	0.714
Lab + tongue + pulse	0.888	0.942	0.810	0.810	0.815	0.809	0.652	0.714
Lab + face + pulse	0.845	0.953	0.817	0.691	0.720	0.765	0.678	0.645
Lab + tongue + face + pulse	0.940	0.964	0.884	0.905	0.811	0.863	0.799	0.803

### 4.7 Fusion results of different decision-making layers

As shown in Table 7, the Adaptive method performed the best on the internal dataset with AUC of 0.940, ACC of 0.964, F1 0.884, and Recall 0.905; on the external dataset, AUC of 0.811, ACC 0.863, F1 0.799 and Recall 0.803 performed relatively stable. In contrast, the MAX method achieved an AUC of 0.938 on the internal dataset, ACC of 0.961 and F1 of 0.915, but its AUC and F1 values decreased to 0.798 and 0.745, respectively. The AUC of the method was 0.905 and ACC 0.957, while the AUC and F1 values on the external dataset were 0.809 and 0.802. The Concat method has an AUC of 0.923 in the internal dataset, but the AUC of its external dataset is 0.796. Adaptive method showed good comprehensive performance on both internal and external data sets.

**TABLE 7:** Fusion results of the different decision layers of the model.

Types	Internal date				External date			
	AUC	ACC	F1	Recall	AUC	ACC	F1	Recall
Concat	0.923	0.828	0.773	0.897	0.796	0.831	0.773	0.803
MAX	0.938	0.961	0.915	0.909	0.798	0.750	0.745	0.903
Mean	0.903	0.942	0.898	0.896	0.834	0.821	0.898	0.803
SUM	0.893	0.942	0.892	0.923	0.887	0.821	0.817	0.6122
Attention	0.905	0.957	0.901	0.835	0.809	0.851	0.802	0.897
Adaptive	0.940	0.964	0.884	0.905	0.811	0.863	0.799	0.803

### 4.8 Model heat map is presented

#### 4.8.1 Pulse thermal map

**FIGURE 11:**
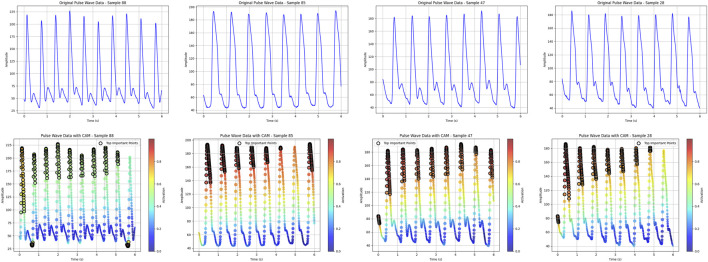
Pulse pressure-wave heat map.

As shown in Figure 11, the top 20% regions of the model focus are marked with black dots. In the data extraction of pulse pressure waves, the model focuses on the region of the main wave h1, focusing on the peak region of the h1 main wave.

#### 4.8.2 Face image heat map

**FIGURE 12:**
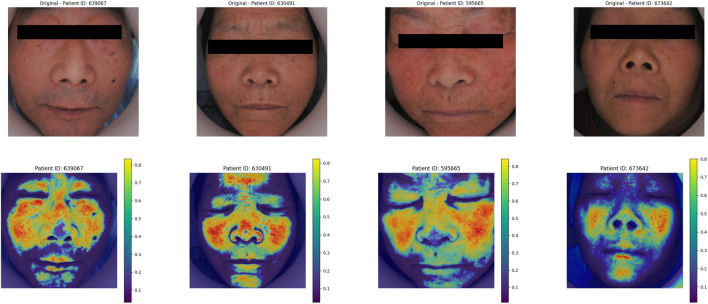
Model of the complexion heat map.

As shown in Figure 12, in the deep learning face image Grad_CAM thermal map, the model focus area was focused on the frontal, nasal and zygomatic regions of the patient.

#### 4.8.3 Thermal map of the tongue image

**FIGURE 13:**
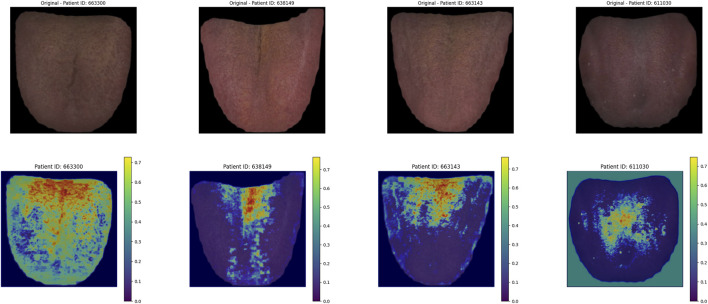
Model tongue thermal map.

As shown in Figure 13, in the deep learning tongue Grad_CAM thermal map, the model focuses on the tongue coating area of the tongue.

## 5 Discussion

An artificial intelligence-based non-invasive predictive approach can serve as an alternative to angiographic results in the diagnosis of coronary artery disease and assist in determining the degree of vascular blockage. This not only reduces the risks associated with exploratory PCI procedures but also minimizes vascular damage to patients, enhancing the safety of diagnosis and treatment. This study predicted the severity of coronary artery stenosis through the fusion of multimodal data. Compared to previous machine learning studies conducted by the team—which used parameter extraction from tongue and facial images in different color spaces and time-domain and value-domain data extraction from pulse wave signals-this model demonstrated superior training accuracy. This indicates that deeper convolutional neural networks can extract richer features from image data (Ling et al., 2019). Additionally, the use of the LSTM model based on time series proved effective in fully capturing the characteristics of pulse wave signals. Unlike existing approaches that focus on time-domain or value-domain features, LSTM allows for a more comprehensive learning of the pulse wave signal, enabling the model to focus on finer details of the waveform over a complete cardiac cycle. Compared to existing non-invasive coronary artery disease risk prediction models, the prediction accuracy of this model showed significant improvement (Park et al., 2019; Lin et al., 2020).

In the data ablation and module ablation experiments, the combination of tongue, face, pulse, and laboratory data yielded the best performance. When analyzing individual data inputs, the combination of laboratory data and pulse wave signals showed the highest predictive capability. Pulse wave data, which reflect the vascular pressure of the radial artery, provide a direct indication of cardiovascular function. The model’s heatmap for pulse wave data revealed that it primarily focused on the peak region of the primary wave, which corresponds to the endpoint of cardiac contraction and the beginning of relaxation. This region may be highly correlated with the degree of vascular stenosis. In the data ablation analysis, the importance of pulse wave data was greater than that of facial features, which in turn was greater than tongue features.

In the module ablation experiments, standalone residual modules or self-attention modules performed suboptimally; however, their combination significantly improved model accuracy. Residual modules enhance the extraction of fine details from deep layers of image data, and the extracted features can then be amplified by the self-attention mechanism (Liu et al., 2020a).

The model’s heatmaps highlight its focus on different modalities. For pulse wave data, the model emphasized arterial compliance and left ventricular ejection function—functions that are directly affected by coronary artery stenosis. The model’s attention to these key features demonstrates its ability to capture the physiological impact of vascular obstruction. For tongue features, the focus was mainly on the tongue coating. Previous studies have suggested a relationship between tongue coating and gut microbiota, with different gut microbiota compositions leading to variations in tongue coating. Gut microbiota is also a major risk factor for coronary artery disease (Liu et al., 2020b; Guo et al., 2022)^.^ For facial image data, the model primarily focused on the forehead, cheekbones, and nose areas. These regions are richly supplied with blood, and coronary artery stenosis can impair the microcirculation in capillary networks. The rich capillary supply in these regions may explain why the model focuses on them (Sanchez-Garcia et al., 2018). Studies have also shown a certain correlation between facial microcirculation and coronary heart disease (Khedkar et al., 2024). The model in this study specifically focused on learning the facial microcirculation region. This suggests that the multimodal transformer model can effectively distinguish different modalities of data through visualization, providing valuable insights into the obstruction of coronary vessels.

This study also addresses the issue of unbalanced patient group sizes. In the experiment, we controlled the sample size of Vascular Obstruction ≥75% to be the same as that of Vascular Obstruction <75% and found that the results were similar to existing findings. Based on considerations regarding sample size, we decided to proceed with the experiment using the current sample size. However, this study has certain limitations. The sample size was relatively small, and larger datasets from multi-center and multi-regional studies would improve the model’s robustness and bring its predictive accuracy closer to real-world performance. Additionally, although uniform equipment was used to collect data in this study—minimizing variability caused by different devices—developing more portable data collection devices would facilitate the clinical application of this model as a diagnostic aid.

## 6 Conclusion

This study develops a multimodal AWCOP model integrating clinical data, tongue images, facial images, and pulse wave data, using adaptive weights for decision-making. The model effectively distinguishes coronary artery blockage.

## Data Availability

The original contributions presented in the study are included in the article/supplementary material, further inquiries can be directed to the corresponding author.
